# Deciphering the Anti-Cancer Efficacy of the Combination of Small-Molecule Inhibitor KAN0438757 and Curcumin in Lung Cancer Cell Lines

**DOI:** 10.3390/cimb47110892

**Published:** 2025-10-28

**Authors:** Deniz Özdemir, Can Ali Ağca

**Affiliations:** 1 Department of Molecular Biology and Genetics, Science Institute, Bingol University, Bingol 12000, Türkiye; 2 Department of Molecular Biology and Genetics, Faculty of Arts and Sciences, Bingol University, Bingol 12000, Türkiye

**Keywords:** KAN0438757, PFKFB3, curcumin, lung cancer

## Abstract

Lung cancer is among the most aggressive malignancies, with the highest incidence and mortality rates worldwide. Standard treatments include surgery, radiotherapy, and chemotherapy; however, chemoresistance often develops, reducing therapeutic efficacy. Combination therapy offers a promising strategy to enhance drug effectiveness and overcome resistance. In lung cancer, the increased energy demands within cells result in a marked rise in the expression of PFKFB3, a regulatory protein involved in the glucose metabolic pathway. The small-molecule inhibitor KAN0438757, recognized as a novel PFKFB3 inhibitor, is significant in targeted therapy due to its essential role in the DNA damage response mechanism in cancer cells. Curcumin, the primary bioactive compound found in the rhizomes of Curcuma longa, has demonstrated a variety of biological functions and anticancer properties. This study aimed to evaluate the anticancer effects of KAN0438757 in combination with curcumin in lung cancer cells. Evaluation of cell viability and IC_50_ values (KAN0438757: A549, 41.13 µM; H1299, 53.74 µM; Curcumin: A549, 44.37 µM; H1299, 66.25 µM) using the WST-1 and RTCA assays revealed pronounced inhibition of proliferation in the combination groups, accompanied by decreased cell migration (fold change, untreated cell; 1, CUR-20 µM; 0.681, KAN-20 µM; 0.530, and COMB; 0.0039 for 48 h). The comet assay revealed severe DNA damage (Tail DNA, fold change, untreated cell; 1, CUR-20 µM; 1.2, KAN-20 µM; 3, and COMB; 4.6) in the A549 cells, while MMP analysis (color change from red to green) and apoptotic staining confirmed cell death morphologically (color change from green to orange). Moreover, Western blot analysis demonstrated that the combination markedly enhanced apoptosis in the A549 cells.

## 1. Introduction

Lung cancer is recognized as one of the most widespread and fatal cancers worldwide, with elevated morbidity and mortality rates primarily associated with risk factors such as tobacco use, environmental pollution, and hereditary predisposition. Current global data reveal that lung cancer constitutes approximately 12.4% of all cancer cases, with nearly 2.5 million new cases reported annually [1]. Non-small cell lung cancer (NSCLC) accounts for approximately 80–85% of all lung cancers, and the 5-year survival rate for NSCLC patients remains below 15% [2]. For decades, the first-line treatment for lung cancer has been chemotherapy, but with limited success. Treatment is often accompanied by severe dose-limiting side effects. Therefore, new administration approaches are being sought to achieve effective treatment with minimal toxicity [3]. Upregulation of glycolysis is one of the main metabolic pathways involved in cancer progression [4]. 6-Phosphofructo-2-kinase/fructose-2,6-bisphosphatases (PFKFBs) are enzymes that exhibit dual kinase and phosphatase activities. The PFKFB family comprises four members (PFKFB1, PFKFB2, PFKFB3, and PFKFB4) [4,5]. Among these, PFKFB3 exhibits higher kinase activity and lower bisphosphatase activity compared with the other isoforms [6], leading to persistent activation of the PFKFB3 gene in lung cancer [7]. High expression of PFKFB3 in lung cancer plays a crucial role in regulating various cellular events including pathological angiogenesis, cell cycle progression, metastasis, and DNA repair [8,9,10]. This indicates that PFKFB3 functions as an oncogene-like regulatory element and that its elevated expression is an independent marker of overall survival in lung cancer patients [5]. Reinforcing the concept of targeting glycolysis through the inhibition of the glycolytic activator PFKFB3, Gustafsson and colleagues recently developed a novel PFKFB3 inhibitor, KAN0438757. Beyond its well-established role in glycolysis, PFKFB3 is also implicated in the repair of DNA double-strand breaks, which are critical for homologous recombination repair [9]. Furthermore, pharmacological inhibition of PFKFB3 by KAN0438757 impairs deoxynucleotide synthesis required for DNA repair, thereby compromising the survival of cancer cells exposed to radiation-induced DNA damage [11]. Curcumin (diferuloylmethane; Cur) is a bioactive polyphenol derived from Curcuma longa (turmeric) with notable pharmacological properties including antioxidant, anti-inflammatory, and anticancer activities [12,13]. Numerous reports have demonstrated that curcumin suppresses cancer cell proliferation in a variety of tumors such as colon, prostate, breast, stomach, lung, liver, and head and neck cancers. It modulates cancer cell behavior by inducing cell cycle arrest, promoting apoptosis, and suppressing angiogenesis [14,15,16,17].

In our previous work, we demonstrated for the first time that KAN0438757, a selective PFKFB3 inhibitor, exhibits potent anticancer activity by markedly reducing the survival and proliferation of A549 and H1299 lung cancer cells and suppressing colony formation and migration in A549 cells. More importantly, KAN0438757 induced DNA damage, mitochondrial membrane potential disruption, and apoptosis, as evidenced by the upregulation of cleaved PARP in A549 cells. Moreover, KAN0438757 treatment led to the upregulation of LC3-II and Beclin-1, suggesting that the compound activates autophagy biomarkers [18]. Nevertheless, growing evidence suggests that the therapeutic efficacy of molecular inhibitors can be enhanced when combined with natural bioactive compounds. Curcumin, a well-studied polyphenolic agent, has been shown to modulate several signaling cascades related to apoptosis, autophagy, and cell cycle arrest. In this context, it remains unclear whether the combination of KAN0438757 and curcumin could exert stronger in vitro anticancer effects than single-agent treatment such as a more pronounced decrease in cell viability and proliferation, greater suppression of migration, increased DNA damage, and enhanced apoptosis. Therefore, we hypothesize that this combinatorial strategy may not only amplify the antitumor potential of KAN0438757, but also highlight the therapeutic promise of combination-based approaches in lung cancer treatment. In this study, we aimed to achieve enhanced anticancer efficacy by combining KAN0438757 with the non-toxic natural compound curcumin, thereby enabling the use of lower doses of KAN0438757 while maintaining or even improving therapeutic effectiveness.

## 2. Materials and Methods

### 2.1. Materials

Ethidium bromide solution (CAS NO:1239-45-8) and acridine orange solution (CAS NO:65-61-2) was obtained from Sigma-Aldrich company (St. Louis, MO, USA). Roswell Park Memorial Institute (RPMI) 1640 medium, fetal bovine serum (FBS), penicillin/streptomycin, and phosphate-buffered saline (PBS) were purchased from Gibco (Waltham, MA, USA). The PFKFB3 inhibitor KAN0438757 (Cat No: S0400) was purchased from Selleck Chemicals GmbH (Houston, TX, USA) and dissolved in sterile dimethyl sulfoxide (DMSO, sterile, suitable for cell culture) to a final concentration of 1 mM. It was then stored at −80 °C and diluted in culture medium to working concentrations.

### 2.2. Cell Culture Conditions

Non-small cell lung cancer cell lines A549 and H1299 were obtained from ATCC (American Type Culture Collection). The main content of the medium used in the culture of A549 and H1299 cells was RPMI 1640 basal medium, 10% fetal bovine serum (FBS), and 1% penicillin/streptomycin. Cells were cultured in sterile medium and grown in a carbon dioxide incubator (5% CO_2_ and 37 °C). The cell culture conditions used in all experimental sets were the same as those used in our previous preclinical study to determine KAN0438757 efficacy [18].

### 2.3. Cell Viability Test

The cytotoxicity assays for KAN0438757 were previously reported in our study [18]. In the current work, the same dose ranges were repeated in combination with curcumin to evaluate the effects on cell viability (KAN0438757 (0, 3, 5, 10, 20, 25, 30, 40 µM) was combined with curcumin (10, 20, 40 µM) doses). For consistency and comparability, all controls were re-assayed under identical conditions. Viability of A549 and H1299 cells was assessed using the WST-1 Assay Kit (Boster, Pleasanton, CA, USA). Cells were seeded (3 × 10^3^ cells per well) in 96-well plates, and treatment was performed after 24 h of incubation. At the end of the treatment period, 10 µL of WST-1 solution was added to each well and incubated for 4 h at 37 °C in a CO_2_ incubator. After incubation, the result of WST-1 at a 450 nm wavelength was determined by measuring absorbance using a microplate reader (Molecular Devices LLC, San Jose, CA, USA) [19]. The cell viability was determined by: % cell viability = absorbance of test group/absorbance of background control × 100 formula. Absorbance values were normalized to the control group, and concentration-dependent cell viability percentages and IC_50_ (half-maximal inhibitory concentration) values were determined by nonlinear regression analysis using GraphPad Prism (version 10.0).

### 2.4. Evaluation of Real-Time Cell Proliferation

The effects of the KAN0438757 and curcumin combination on cell proliferation in A549 cell lines, separately, were determined using the xCELLigence Real-Time Cell Analyzer (ACEA, San Diego, CA, USA). Cells were seeded into 8-well RTCA-E cell culture plates at 1 × 10^10^ cells per well. Cells were treated following the protocol developed by the manufacturer. The program was set to record data every 15 min and the CI (cell index) graph was obtained [20].

### 2.5. In Vitro Scratch Assay

Cell migration was examined using the wound scratch assay. Cells were seeded in 6-well culture plates for their adhesion. After 24 h of incubation, confluent monolayer cells were washed. A mechanical scratch was made vertically with DPBS and with the aid of a 200 µL pipette tip to create fixed diameter strips. Residue and detached cells were washed with DPBS buffer and then placed in fresh serum-free RPMI 1640/medium. Wound areas were observed on images taken with an inverted microscope and photographed using an inverted microscope at intervals of 0, 6, 24, 30 and 48 h. Wound closure was measured with the ImageJ/Wound Healing Size Tool (Version 1.50i). Three random fields per well were analyzed. The experiment was repeated at least three times [21].

### 2.6. Mitochondrial Membrane Potential (MMP) Assay

In the logarithmic growth phase, 2 × 10^5^ cells per well were seeded onto glass-bottomed culture plates. Treatment was then continued for 48 h. The plates were washed and stained with the cationic dye JC-1 (2 µM) for 15 min at 37 °C. The staining change was analyzed using a microscope (Olympus, Tokyo, Japan). The experiment was repeated three times. Total count and total area were analyzed with ImageJ (Version 1.50i). Color thresholding was used for area calculation [22].

### 2.7. Acridine Orange and Ethidium Bromide Double Staining

Fluorescence-based determination of apoptosis in A549 and H1299 cells was performed using the acridine orange and ethidium bromide (AO/EtBr) staining method as described by Baskic et al. After the lung cancer cells were incubated with KAN0438757 (20 µM), curcumin (20 µM), and KAN0438757 (20 µM) + curcumin (20 µM) for 48 h, the cells were harvested, washed with PBS, and stained with AO/EtBr (100 μL of PBS, 10 μg/mL of AO and 10 μg/mL of PI). Cells were photographed under a fluorescence microscope and compared with the control. The nuclei of living cells are stained green only due to the permeability of acridine orange, while apoptotic cells appear red/orange due to co-staining of both fluorescent dyes [23]. The percentage of viable, early apoptotic, late apoptotic, and necrotic cells was calculated from at least 20 cells per sample using ImageJ software (Version 1.50i). Results are expressed as the mean ± SD of three independent experiments.

### 2.8. Single Cell Gel Electrophoresis (Comet Assay)

A549 cells were seeded in 6-well plates (2 × 10^5^ cells per well) and treated with the indicated doses of KAN0438757 (20 µM), curcumin (20 µM), and the combination of KAN0438757 (20 µM) + curcumin (20 µM) for 48 h. At the end of the treatment period, the wells were washed with PBS, and the cells were dissociated using trypsin. The cells were then centrifuged, resuspended in PBS, and mixed with 0.5% low-melting-point agarose (LMA). Subsequently, 20 µL of this mixture was spread onto a slide precoated with normal-melting-point agarose (NMA). The slides were covered with coverslips and kept at 4 °C for 45 min. After incubation, the coverslips were removed, and the slides were placed in cold lysis solution (2.5 M NaCl, 100 mM EDTA, 10 mM Tris, 10% DMSO, and 1% Triton X-100, pH 10) for 60 min at 4 °C. The slides were then rinsed with PBS and transferred to an electrophoresis tank containing a neutral buffer. Electrophoresis was performed at 28 V and 300 mA for 25 min (pH 7.5), after which the slides were rinsed again with PBS. The slides were stained with ethidium bromide (10 µg/mL) for 15 min and examined under a fluorescence microscope. For each experimental group, at least 50 randomly selected cells from three independent experiments (n = 3) were analyzed and quantified using the Comet Score software program (Version 2.0) [24].

### 2.9. Cell Lysate Preparation and Western Blot Analysis

After the treatment, the cells in the culture dishes were removed with the help of a scraper after washing with cold PBS and transferred to microcentrifuge tubes in PBS. Cells were centrifuged at 6000 rpm for 10 min and the supernatant was aspirated and removed. The pellet was then suspended in cold RIPA lysis buffer to which protease inhibitors were added and incubated on ice for 45 min. After the incubation period, it was centrifuged for 10 min at 15,000 rpm at 4 °C. The obtained supernatant was transferred to another tube and the total protein concentrations of the samples were determined at 595 nm by the Bradford method. With the help of the electrophoresis system (Bio-Rad Trans-Blot cell, BioRad, Hercules, CA, USA), protein samples and markers were fractionated on SDS-PAGE (10–12%) gel. Then, the proteins separated in the gel were transferred to a polyvinylidene difluoride (PVDF) membrane. After the transfer process, the PVDF membrane was incubated in 5% skim milk powder at room temperature for one hour. Subsequently, the PVDF membrane was incubated with the relevant primary antibody (in the dilution ratios recommended by the manufacturer) at +4 °C overnight on a shaker. After the incubation, the membrane was washed 5 min/5 times with 1X TBS-T and incubated with secondary antibody compatible with the primary antibody (at the dilution rates recommended by the manufacturer) for 60 min at room temperature. Images were obtained using the membrane ECL chemiluminescent imaging kit. The intensities of the bands of the respective proteins were evaluated densitometrically using the ImageJ program (Image J; National Institute of Health, Bethesda, MD, USA). Primer antibodies (anti-GAPDH, Santa Cruz, Santa Cruz, CA, USA, sc-365062, 1:1000), TIGAR (Santa Cruz, sc-377065, 1:1000), Bax (Abcam, Cambridge, UK, ab7977, 1:1000), and secondary antibodies (anti-mouse IgG-HRP, Jackson, West Grove, PA, USA, 115-035-166, 1:5000), and anti-rabbit IgG-HRP, sc-2357, 1:5000) were used. Densitometrical readings of the bands were normalized according to GAPDH expression [25].

### 2.10. Statistical Analysis

Statistical analyses were performed using GraphPad Prism software (Prism 10, GraphPad Software Inc., La Jolla, CA, USA). Data were presented as the mean ± standard deviation (SD) of at least three experiments. Comparisons between the control and treatment groups were made using one-way ANOVA and Tukey’s multiple comparison tests. The level of statistical significance was considered based on the *p* value (* *p* < 0.05; ** *p* < 0.01; *** *p* < 0.001; **** *p* < 0.0001).

## 3. Results

### 3.1. Antiproliferative Effects of KAN0438757, Curcumin, and Their Combination in A549 and H1299 Cells

**Figure 1:**
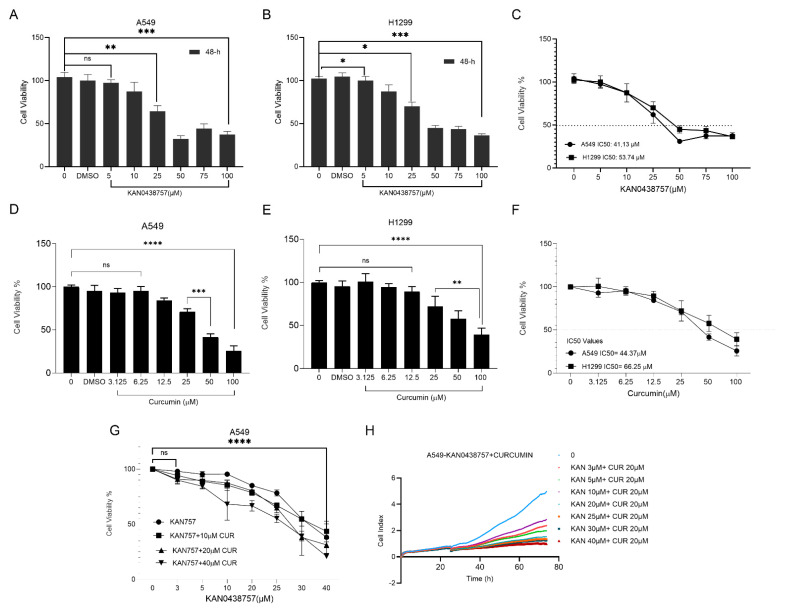
The combination of KAN0438757 and curcumin chemotherapy effectively suppresses the growth of human lung cancer cells. Human lung cancer cell lines A549 (**A**–**C**,**G**) and H1299 (**D**–**F**) were subjected to various concentrations of KAN0438757 alone, curcumin alone, or a combination of KAN0438757 and curcumin for a duration of 48 h, after which the cell viability was assessed using the WST-1 assay. Both KAN0438757 alone (**A**–**C**) and curcumin alone (**D**–**F**) as well as their combination (**G**) demonstrated dose-dependent inhibitory effects on lung cancer cell viability. The combination treatments exhibited greater efficacy compared with the individual drug treatments in both cell lines. The values are expressed as the mean ± SD (n = 3/group). IC_50_ values of KAN0438757 and curcumin in the A549 and H1299 cell lines. (H) The combination of KAN0438757 and curcumin reduced the proliferation in the A549 cell line. The results were obtained by one-way ANOVA test (* *p* < 0.05; ** *p* < 0.01; *** *p* < 0.001; **** *p* < 0.0001, ns = not significant).

We detected the first cytotoxicity study of KAN0438757 as a single-agent treatment in A549 and H1299 lung cancer cell lines in a previous experimental study [18]. In this study, the effects of A549 and H1299 were investigated to examine whether the combination of KAN0438757 and curcumin exhibited anti-proliferative activity. The effects on cell viability were determined using the WST-1 test, according to the manufacturer’s protocol. Lung cancer cells (A549 and H1299) were treated with various concentrations of KAN0438757 and curcumin for 48 h. In addition, A549 cells were treated with KAN0438757 and curcumin combinations for 48 h. As seen in Figure 1A–C, both cell lines of KAN0438757 were significantly decreased in a dose-dependent manner during the treatments. The half-maximum inhibitory concentration (IC_50_) is the numerical value of the dose that inhibits the cell viability of half of the cells. The IC_50_ values of KAN0438757 were determined as follows: IC_50_ value in the A549 cell line at 41.13 µM and the IC_50_ in H1299 was determined at 53.74 µM. The results obtained were consistent with our previous study [18]. Curcumin was treated at different concentrations and the A549 IC_50_ value of curcumin determined in the A549 cell line was 44.37 µM. The IC_50_ value detected in the H1299 cell line was calculated as 66.25 µM. As can be seen in the graph in Figure 1D–F, curcumin showed a very high effect in two different cell lines in a dose-dependent manner compared with the control group. As seen in the graph in Figure 1G, the A549 cells showed a very high effect of the combination in a dose-dependent manner compared with the control group. Cell index was determined by Xcelligence to evaluate its effect on cell proliferation following combination treatment in the A549 cell line, are shown in Figure 1H. It was determined that the cell proliferation decreased in a dose-dependent manner in the A549 cell line. This study primarily focused on the A549 cell line to investigate the anti-cancer effects of the combination of KAN0438757 and curcumin. Further validation in additional NSCLC cell lines will be required to generalize these findings.

### 3.2. Cell Migration Findings of KAN0438757 and Curcumin Combinations in A549 Cell Line

**Figure 2:**
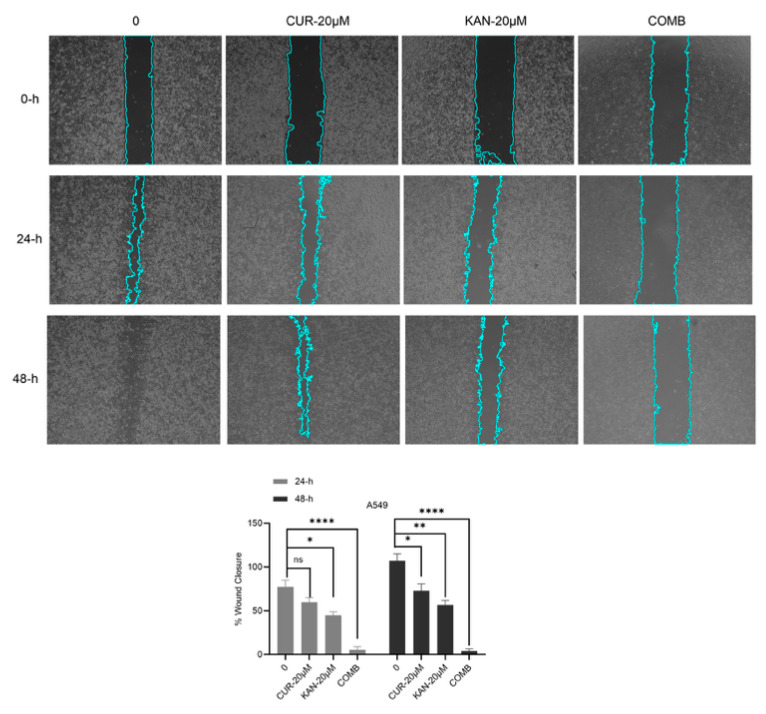
KAN0438757 and curcumin combinations were found to inhibit the capability of cell migration. Wound healing assay was executed at 0, 24, and 48 h post-treatment of the A549 cell line with KAN0438757 and curcumin combinations, observed under a microscope (original magnification 4×). A scratch was created in the cells in the Petri dish, starting from the central area, and images were taken under a microscope (500 µm scale bar) to evaluate whether the scratch would close over 48 h, thus assessing the cells’ migratory ability. The wound closure in the A549 cells was analyzed using microscope images captured at 24 and 48 h. The values are expressed as the mean ± SD (n = 3/group). The results were obtained by one-way ANOVA (* *p* < 0.05; ** *p* < 0.01; **** *p* < 0.0001; ns = not significant).

To determine the effects of the KAN0438757 and curcumin combination on cell migration, a wound healing assay was performed on the A549 cells. As shown in Figure 2, it significantly reduced the migration ability of the cells of the combination group and stopped cell proliferation. On the other hand, a comparatively slow migration and invasion occurred in the KAN0438757-20 µM and curcumin-20 µM groups, with promising results in the combination group, where it was determined that KAN0438757 and curcumin may have an inhibitory effect on cell migration. A significant change was observed compared with the control group, as shown in Figure 2.

### 3.3. DNA Damage in A549 Cells Induced by KAN0438757–Curcumin Combination

**Figure 3:**
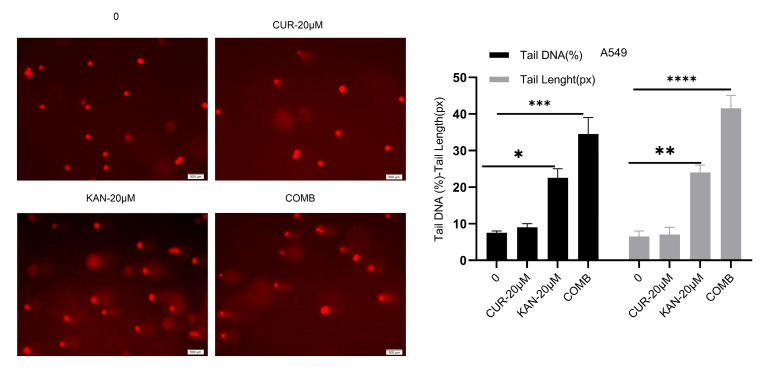
Comet test on lung cancer cells treated for 48 h with a combination of KAN0438757 and curcumin. Cells on the combination treatment showed a significant difference in their length. The values are expressed as the mean ± SD (n = 3/group). The results were obtained by one-way ANOVA (* *p* < 0.05; ** *p* < 0.01; *** *p* < 0.001; **** *p* < 0.0001).

To evaluate the influence of KAN0438757 and curcumin combinations on DNA damage in A549, DNA damage was analyzed through single-cell gel electrophoresis. While no tail formation was observed in the control and curcumin treatment groups, increased DNA damage was observed to cause significant tail formation in the KAN and combination groups. Statistical significance was observed when compared with the control group. KAN0438757 was determined to increase the effectiveness of curcumin (Figure 3).

### 3.4. Mitochondrial Membrane Potential and Cell Death in A549 Cells Treated with KAN0438757–Curcumin Combination

**Figure 4:**
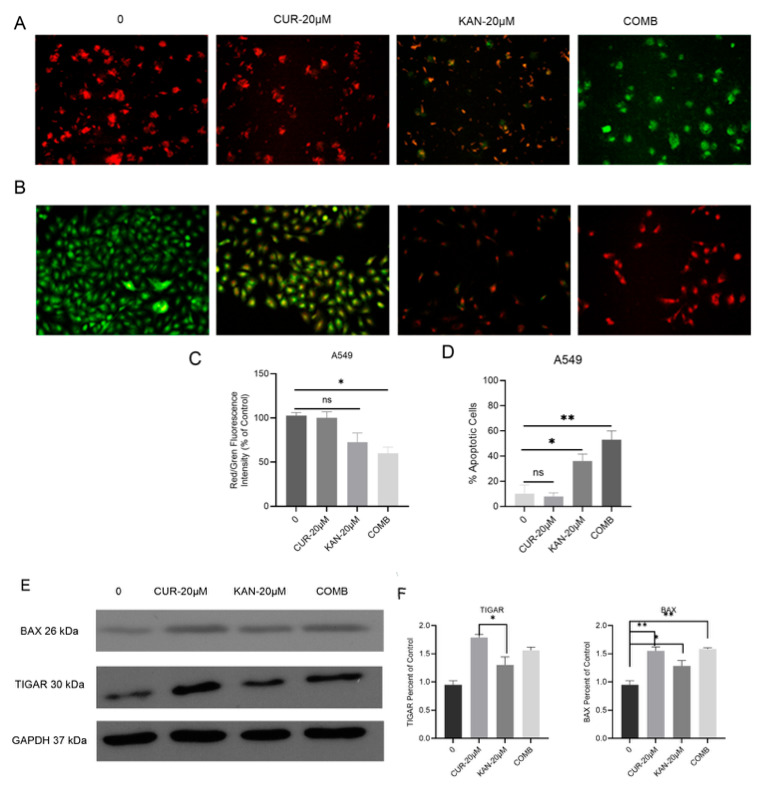
Effects of KAN0438757 and curcumin combination on mitochondrial membrane potential (MMP) and cell death in A549 cells. (**A**,**C**) JC-1 staining shows the mitochondrial membrane depolarization in A549 cells treated with the KAN0438757 and curcumin combination for 48 h. Images were captured using a live imaging microscope (OLYMPUS CKX41) at × 20 magnification. The loss of ΔΨm was evaluated using JC-1 dye, and fluorescence was recorded at 530/590 nm. Results are shown as the mean ± SD from triplicate samples. (**B**,**D**,**E**) Apoptosis analysis by acridine orange/ethidium bromide (AO/EB) staining. In cells treated with 20 µM curcumin alone, no apparent color change was observed, indicating limited apoptotic activity. Treatment with 20 µM KAN0438757 alone induced noticeable cell death, while the combination of curcumin and KAN0438757 further enhanced apoptosis. (**F**) The effects of KAN0438757 and curcumin combinations on the expression levels of TIGAR and Bax proteins, with GAPDH used as a loading control. Statistical significance was determined using one-way ANOVA followed by Tukey’s multiple comparison test (GraphPad Prism 10.0, GraphPad Software Inc.). (* *p* < 0.05; ** *p* < 0.01; ns = not significant).

The anti-cancer effects of the combination of KAN0438757 and curcumin on MMP and apoptosis were evaluated. As seen in Figure 4A, a color change from green to red occurred in the KAN-20 µM and COMB treatment groups, which was considered to indicate the disruption of mitochondrial membrane integrity. A significant change was observed in the COMB group when the red/green fluorescence intensity analysis was examined (Figure 4C). Another experimental result, as shown in Figure 4B, showed an apoptotic morphology: green-stained nuclei indicate viable cells, greenish-yellow nuclei indicate early apoptotic cells, and intensely red nuclei indicate late apoptotic cells. In the KAN-20 µM and COMB treatments, the color of the cells changed to orange and yellow during staining, indicating that the cells approached the apoptotic phase in a dose-dependent manner with increasing doses. Statistical analysis was performed by measuring the red and green fluorescence intensities, and a significant change was observed (Figure 4D). The expression levels of the proteins determined by Western blot analysis are shown in Figure 4E. Significant increases in the expression of the proapoptotic Bax protein were observed compared with the control group. A significant change in the TIGAR protein was noted in the KAN-20 µM treatment group compared with the CUR-20 µM group (Figure 4F).

## 4. Discussion

Cancer is a disease characterized by the uncontrolled division and proliferation of certain cells that invade normal tissues. Lung cancer is the leading cause of cancer-related death, responsible for approximately 1.38 million deaths annually, which exceeds the combined mortality rates of breast, colorectal, and prostate cancers [26]. Currently, chemotherapy yields a median survival rate of only about 35% at one year and approximately 15% at two years [27]. Cancer cells exhibit increased glycolysis even in the presence of oxygen, a phenomenon known as the “Warburg effect” [28]. Glucose is considered the preferred energy and biosynthetic substrate for rapidly growing tumor cells [29]. Fructose-2,6-bisphosphate (F26BP) is an allosteric activator of PFK-1 and is regarded as a key factor in glycolytic control. Moreover, PFKFB3 is frequently overexpressed in lung, breast, and prostate tumors. High PFKFB3 expression is associated with poor survival, metastasis, and dysregulation of the cell cycle and apoptosis [30,31]. Several PFKFB3 inhibitors have been identified including 3PO, PFK15, and PFK158. A novel small-molecule PFKFB3 inhibitor, KAN0438757, was recently reported [9]. Curcumin, derived from Curcuma longa, exhibits a wide range of pharmacological activities including antioxidant, antiviral, antifungal, antibacterial, and anticancer effects [32]. The effect of the KAN0438757 molecule on the A549 and H1299 cell lines was evaluated using the WST-1 cell viability assay. The results demonstrated significant cell death at doses ranging from 10 to 50 µM in both cell lines. When the IC_50_ values of KAN0438757 in the A549 and H1299 cells were compared, they were found to be lower in A549 (41.13 µM) than in H1299 (53.74 µM). In a study using the PFKFB3 inhibitor PFK158 in endometrial cell lines, cell viability was reported to decrease gradually in a dose-dependent manner [33]. Oliveira et al. (2021) showed that treatment with KAN0438757 reduced the cell viability in a concentration-dependent manner in the HCT-116, SW-1463, and HUVEC cells [11]. In our study, curcumin exhibited concentration-dependent cytotoxic effects in both A549 and H1299 lung cancer cells, with IC_50_ values measured at 44.37 µM and 66.25 µM, respectively. These findings are consistent with previous reports by Baharuddin et al. (2016) [34] and Pillai et al. (2004) [35]. Consistent with our prior findings [18], the KAN0438757 single-agent treatment reduced viability in lung cancer cells. In this study, the combination with curcumin further enhanced the effect. A dose-dependent decrease in cell proliferation was also observed in a simultaneous cell proliferation assay using the combined KAN0438757 and curcumin treatment in A549 cells. Consistently, treatment with KAN0438757 in combination with curcumin resulted in a markedly stronger antiproliferative effect than that observed with single-agent treatment in our previous study [18]. KAN0438757 appears to possess high efficacy by enhancing the effectiveness of curcumin. In real-time cell profiling experiments, Oliveira et al. (2021) [11] observed the most potent effect of KAN0438757 alone in three different cell lines (HCT116, HT19, and HUVEC). They reported that cell proliferation progressively ceased in the 25, 50, and 75 µM treatment groups, highlighting the compound’s strong efficacy.

Cell migration is defined as the directed movement of single cells or cell groups in response to intracellular signals [21]. In the combined curcumin and KAN0438757 treatment study, the cell migration assay revealed that intercellular spaces closed most rapidly in the control group, while the KAN-20 µM + Cur-20 µM group showed minimal closure. We previously demonstrated the inhibitory effect of KAN0438757 single-agent treatment on cell migration in A549 cells [18]. However, the combination of KAN0438757 with curcumin exhibited a stronger inhibitory effect on cell migration. The wound healing assay performed by Oliveira et al. (2021) demonstrated that 25 µM KAN0438757 treatment in HCT116, HT19, and HUVEC cells did not close intercellular spaces compared with the control groups [11].

DNA damage plays an essential role in determining the effectiveness of chemotherapeutic agents. In our previous study, we showed that KAN0438757 induced DNA damage in a dose-dependent manner in the A549 lung cancer cell line; however, the current study demonstrated for the first time that the combination of KAN0438757 and curcumin produced a greater effect compared with single-agent treatment [18]. In single-cell gel electrophoresis (comet) assays, the combination of KAN0438757 and curcumin was statistically identified as the group exhibiting the highest level of DNA damage. Smaller tail formation was observed in the KAN0438757 (20 µM) group compared with the combination group, indicating the high efficacy of the combination treatment. Gustafsson et al. demonstrated that KAN0438757, beyond its role in glycolysis, plays a critical role in repairing DNA double-strand breaks through homologous recombination [9]. Acridine orange–ethidium bromide staining showed that the combination of KAN0438757 and curcumin in A549 cells induced the highest late apoptosis compared with KAN0438757 (20 µM) or curcumin (20 µM) alone, with statistical significance relative to the control. In our previous experimental study, KAN0438757 alone induced significant apoptosis in the A549 cell line [18]. On the other hand, it was not known what kind of effect the combinatory effect had, so it was seen that the combination of KAN0438757 and curcumin had an anti-cancer effect in the A549 cell line. Similarly, Lypova et al. (2021) reported that 5 µM PFK158 alone triggered early apoptosis in PC9 cells [33]. Mitochondrial membrane potential (MMP, Δψm), a key indicator of mitochondrial function and cell viability, decreases as integrity is lost and pro-apoptotic signaling is initiated [36]. In our study, reductions in cell density and color changes were evident in the single-agent groups (KAN0438757, 20 µM and curcumin, 20 µM), but these effects were more pronounced in the combined group. In our previous study, KAN0438757 treatment alone caused changes in MMP in the 10 and 25 µM groups [18]; however, in the current study, the combination of KAN0438757 with curcumin disrupted MMP integrity more extensively in the A549 cells. Klarer et al. (2014) found that 3PO alone activated autophagy in HCT-116 cells [37]. Western blot analysis showed increased TIGAR levels in the combination group compared with the control, with the highest expression observed in the curcumin group. Bax protein was markedly elevated in the combination group, confirming apoptosis consistent with the staining results. These results suggest that the combination of KAN0438757 and curcumin increases apoptosis and DNA damage. While our data are consistent with the potential role of PFKFB3-mediated homologous recombination impairment and curcumin’s modulation of signaling pathways, direct mechanistic evidence is not yet available and will be explored in future studies. Zhu et al. (2016) reported that PFK15 treatment decreased Bcl-2 expression without affecting Bax levels in gastric cancer cells, thereby reducing the Bcl-2/Bax ratio, suggesting that the induction of mitochondrial apoptosis likely involves caspase-9 and caspase-3, but not caspase-8 [38]. Collectively, these results indicate that curcumin and KAN0438757 induce programmed cell death in A549 cells.

## 5. Conclusions

In the light of the data obtained in this study, it is thought that the combination of KAN0438757 and curcumin is significantly effective in the in vitro inhibition of lung cancer cell proliferation and may shed light on future studies.

## Data Availability

The data presented in this study are available on request from the corresponding author.

## Abbreviations

The following abbreviations are used in this manuscript:

PFKFB	6-phosphofructo-2-kinase/fructose-2,6 bisphosphatase
NSCLC	Non-small cell lung cancer
MMP	Mitochondrial membrane potential
PFKFB3	6-phosphofructo-2-kinase/fructose-2,6-bisphosphatase 3
Curcumin	Diferuloylmethane; Cur
DMSO	Dimethyl sulfoxide
RTCA	Real-Time Cell Analyzer
FBS	Fetal Bovine Serum
CI	Cell Index
NMA	Normal-Melting-Point Agarose
LMA	Low-Melting-Point Agarose
AO/EtBr	Acridine Orange and Ethidium Bromide
PVDF	Polyvinylidene Difluoride
IC_50_	Half-Maximal Inhibitory Concentration

## References

[1] Yu X., Jia L., Tang Q., Zhou Q., Wang G., Wang S. (2025). Regulation of cisplatin resistance in lung cancer by epigenetic mechanisms. Clin. Epigenetics.

[2] Hirsch F.R., Scagliotti G.V., Mulshine J.L., Kwon R., Curran W.J., Wu Y.-L., Paz-Ares L. (2017). Lung cancer: Current therapies and new targeted treatments. Lancet.

[3] Tiseo M., Bartolotti M., Gelsomino F., Ardizzoni A. (2009). First-line treatment in advanced non-small-cell lung cancer: The emerging role of the histologic subtype. Expert Rev. Anticancer Ther..

[4] Shi L., Pan H., Liu Z., Xie J., Han W. (2017). Roles of PFKFB3 in cancer. Signal Transduct. Target. Ther..

[5] Li X., Liu J., Qian L., Ke H., Yao C., Tian W., Zhang J. (2018). Expression of PFKFB3 and Ki67 in lung adenocarcinomas and targeting PFKFB3 as a therapeutic strategy. Mol. Cell. Biochem..

[6] Clem B., Telang S., Clem A., Yalcin A., Meier J., Simmons A., Chesney J. (2008). Small-molecule inhibition of 6-phosphofructo-2-kinase activity suppresses glycolytic flux and tumor growth. Mol. Cancer Ther..

[7] Lypova N., Telang S., Chesney J., Imbert-Fernandez Y. (2019). Increased 6-phosphofructo-2-kinase/fructose-2, 6-bisphosphatase-3 activity in response to EGFR signaling contributes to non–small cell lung cancer cell survival. J. Biol. Chem..

[8] Jia W., Zhao X., Zhao L., Yan H., Li J., Yang H., Liu J. (2018). Non-canonical roles of PFKFB3 in regulation of cell cycle through binding to CDK4. Oncogene.

[9] Gustafsson N.M., Färnegårdh K., Bonagas N., Ninou A.H., Groth P., Wiita E., Helleday T. (2018). Targeting PFKFB3 radiosensitizes cancer cells and suppresses homologous recombination. Nat. Commun..

[10] Cantelmo A.R., Conradi L.C., Brajic A., Goveia J., Kalucka J., Pircher A., Carmeliet P. (2016). Inhibition of the glycolytic activator PFKFB3 in endothelium induces tumor vessel normalization, impairs metastasis, and improves chemotherapy. Cancer Cell.

[11] De Oliveira T., Goldhardt T., Edelmann M., Rogge T., Rauch K., Kyuchukov N.D., Conradi L.C. (2021). Effects of the novel pfkfb3 inhibitor kan0438757 on colorectal cancer cells and its systemic toxicity evaluation in vivo. Cancers.

[12] Alsamydai A., Jaber N. (2018). Pharmacological aspects of curcumin: Review article. Int. J. Pharmacogn..

[13] Liu F., Gao S., Yang Y., Zhao X., Fan Y., Ma W., Yu Y. (2018). Antitumor activity of curcumin by modulation of apoptosis and autophagy in human lung cancer A549 cells through inhibiting PI3K/Akt/mTOR pathway. Oncol. Rep..

[14] Panzarini E., Mariano S., Tacconi S., Carata E., Tata A.M., Dini L. (2021). Novel therapeutic delivery of nanocurcumin in central nervous system related disorders. Nanomaterials.

[15] Aggarwal B.B., Kumar A., Bharti A.C. (2003). Anticancer potential of curcumin: Preclinical and clinical studies. Anticancer Res..

[16] Mehta H.J., Patel V., Sadikot R.T. (2014). Curcumin and lung cancer—A review. Target. Oncol..

[17] Wan Mohd Tajuddin W.N.B., Lajis N.H., Abas F., Othman I., Naidu R. (2019). Mechanistic Understanding of Curcumin’s Therapeutic Effects in Lung Cancer. Nutrients.

[18] Deniz Ö., Saruhan S., Ağca C.A. (2023). Kan0438757: A Novel Pfkfb3 Inhibitor That Induces Programmed Cell Death and Suppresses Cell Migration in Non-Small Cell Lung Carcinoma Cells. Biotechnol. Acta.

[19] Çağlar H.O., Süslüer S.Y., Kavaklı Ş., Gündüz C., Ertürk B., Özkınay F., Haydaroğlu A. (2017). Meme kanseri kök hücrelerinde elajik asit ile indüklenmiş miRNA’ların ifadesi ve elajik asidin apoptoz üzerine etkisi. Ege Tıp Derg..

[20] Hamidi H., Lilja J., Ivaska J. (2017). Using xCELLigence RTCA Instrument to Measure Cell Adhesion. Bio-Protoc..

[21] Cory G. (2011). Scratch-wound assay. Cell Migration: Developmental methods and protocols.

[22] Kasibhatla S., Amarante-Mendes G.P., Finucane D., Brunner T., Bossy-Wetzel E., Green D.R. (2006). Acridine orange/ethidium bromide (AO/EB) staining to detect apoptosis. Cold Spring Harb. Protoc..

[23] Özdemir D., Ağca C.A. (2025). AZD1390, an Ataxia telangiectasia mutated inhibitor, enhances cisplatin mediated apoptosis in breast cancer cells. Exp. Cell Res..

[24] Agca C.A., Kırıcı M., Nedzvetsky V.S., Gundogdu R., Tykhomyrov A.A. (2020). The Effect of TIGAR Knockdown on Apoptotic and Epithelial-Mesenchymal Markers Expression in Doxorubicin-Resistant Non-Small Cell Lung Cancer A549 Cell Lines. Chem. Biodivers..

[25] World Health Organization (2018). WHO Global Report on Trends in Prevalence of Tobacco Smoking 2000–2025.

[26] Pendharkar D., Ausekar B.V., Gupta S. (2013). Molecular Biology of Lung Cancer-A Review. Indian J. Surg. Oncol..

[27] Warburg O., Wind F., Negelein E. (1927). The Metabolism of Tumors in the Bod. J. Gen. Physiol..

[28] Cao Y., Zhang X., Wang L., Yang Q., Ma Q., Xu J., Huo Y. (2019). PFKFB3-mediated endothelial glycolysis promotes pulmonary hypertension. Proc. Natl. Acad. Sci. USA.

[29] Pavlova N.N., Thompson C.B. (2016). The emerging hallmarks of cancer metabolism. Cell Metab..

[30] Ros S., Schulze A. (2013). Balancing glycolytic flux: The role of 6-phosphofructo-2-kinase/fructose 2, 6-bisphosphatases in cancer metabolism. Cancer Metab..

[31] Pandey A., Gupta R.K., Srivastava R. (2011). Curcumin-the yellow magic. Asian J. Appl. Sci..

[32] Xiao Y., Jin L., Deng C., Guan Y., Kalogera E., Ray U., Shridhar V. (2021). Inhibition of PFKFB3 induces cell death and synergistically enhances chemosensitivity in endometrial cancer. Oncogene.

[33] Lypova N., Dougherty S.M., Lanceta L., Chesney J., Imbert-Fernandez Y. (2021). PFKFB3 Inhibition Impairs Erlotinib-Induced Autophagy in NSCLCs. Cells.

[34] Baharuddin P., Satar N., Fakiruddin K.S., Zakaria N., Lim M.N., Yusoff N.M., Yahaya B.H. (2016). Curcumin improves the efficacy of cisplatin by targeting cancer stem-like cells through p21 and cyclin D1-mediated tumour cell inhibition in non-small cell lung cancer cell lines. Oncol. Rep..

[35] Pillai G.R., Srivastava A.S., Hassanein T.I., Chauhan D.P., Carrier E. (2004). Induction of apoptosis in human lung cancer cells by curcumin. Cancer Lett..

[36] Zorova L.D., Popkov V.A., Plotnikov E.Y., Silachev D.N., Pevzner I.B., Jankauskas S.S., Zorov D.B. (2018). Mitochondrial membrane potential. Anal. Biochem..

[37] Klarer A.C., O’Neal J., Imbert-Fernandez Y., Clem A., Ellis S.R., Clark J., Telang S. (2014). Inhibition of 6-phosphofructo-2-kinase (PFKFB3) induces autophagy as a survival mechanism. Cancer Metab..

[38] Zhu W., Ye L., Zhang J., Yu P., Wang H., Ye Z., Tian J. (2016). PFK15, a small molecule inhibitor of PFKFB3, induces cell cycle arrest, apoptosis and inhibits invasion in gastric cancer. PLoS ONE.

